# Validity and Efficacy of Methods to Define Blood Brain Barrier Integrity in Experimental Ischemic Strokes: A Comparison of Albumin Western Blot, IgG Western Blot and Albumin Immunofluorescence

**DOI:** 10.3390/mps4010023

**Published:** 2021-03-23

**Authors:** Maximilian Franke, Michael Bieber, Guido Stoll, Michael Klaus Schuhmann

**Affiliations:** Department of Neurology, University Hospital Wuerzburg, Josef-Schneider-Str. 11, 97080 Würzburg, Germany; franke_m1@ukw.de (M.F.); bieber_m@ukw.de (M.B.); stoll_g@ukw.de (G.S.)

**Keywords:** IgG, albumin, immunohistochemistry, Western blot, stroke, tMCAO, blood brain barrier, neuroinflammation

## Abstract

The clinical and preclinical research of ischemic strokes (IS) is becoming increasingly comprehensive, especially with the emerging evidence of complex thrombotic and inflammatory interactions. Within these, the blood brain barrier (BBB) plays an important role in regulating the cellular interactions at the vascular interface and is therefore the object of many IS-related questions. Consequently, valid, economic and responsible methods to define BBB integrity are necessary. Therefore, we compared the three ex-vivo setups albumin Western blot (WB), IgG WB and albumin intensity measurement (AIM) with regard to validity as well as temporal and economic efficacy. While the informative value of the three methods correlated significantly, the efficacy of the IgG WB dominated.

## 1. Introduction

The unique microenvironment within the central nervous system (CNS) relies upon the integrity of the blood-brain barrier (BBB). It plays an indispensable role in regulating the traffic of cells, fluids and solutes at the blood-brain interface. The BBB disruption represents a key pathological feature of ischemic strokes (IS). As a result, endothelial lesions occur, para-cellular and trans-cellular permeability increases, water and ion homeostasis disrupt and infiltrating pro-inflammatory cells, like leukocytes, further exacerbate inflammatory processes and brain injury [1,2,3,4,5].

This is the reason why researchers, acting within the scope of BBB-compromising diseases in general or within the environment of experimental strokes in special, require convenient and reliable techniques to characterize the BBB integrity. Various methods to measure the degree of BBB disruption in murine models exist, both in vivo and ex vivo. To the first group belong the undoubtedly informative, but at the same time labor-intensive, equipment- and infrastructure-dependent as well as expensive live imaging technologies like magnetic resonance imaging (MRI) or computed tomography (CT) with perfusion-sensitive weightings or as single photon emission CT (SPECT) [6,7]. Another well-established live imaging technique to examine BBB integrity is in-vivo two-photon microscopy [8,9]. This technology, though, requires further anesthesia, which might interfere with the superior aims of the respective studies. Commonly used isoflurane narcosis, in particular, is associated with neuroprotective bystander effects [10,11]. When it comes to the determination of the BBB function post mortem, a mainstay has long been the Evans Blue dye technique, in which the blue azo dye, binding to serum albumin, is injected in the jugular or tail vein of the living murine animals. It is posthumously assessed either qualitatively, by a simple visualization of blue discoloration within the sectioned brain tissue, or quantitatively, by incubating the extracted tissue in formamide and measuring the dye concentration with ultraviolet spectrophotometry [6,9,12]. Yet after the aforementioned measurements, the dyed tissue is no longer suitable for further histological or biochemical investigations. Furthermore, growing evidence revealed the toxicity of Evans Blue, with an increased periprocedural mortality of the animals [13,14,15]. With researchers expected to adhere to the *3R-principle* to replace, reduce and refine animal experiments, alternatives are necessary [16].

Potential methods of choice to assess BBB integrity are therefore the IgG and albumin Western blots (WB) of the respective brain lysates or the albumin intensity measurement (AIM) after immunofluorescence staining. The advantage of these methods is their small tissue demand, easy implementation, relatively low costs and the absence of the necessity to apply substances into living animals [17,18,19]. Furthermore, the three methods can be applied spontaneously, even if the determination of the BBB integrity might not have been taken into consideration when predefining an experimental setup.

Even though IgG, albumin WB and AIM are widespread and well established, a clear comparison of the techniques with regard to validity and efficacy has, to our knowledge, not been performed.

## 2. Study Design

Brain sections/tissue of mice after a 60-min transient middle cerebral artery occlusion (tMCAO) had been previously analyzed. The mice had been treated with anti-inflammatory medication or vehicle before stroke induction. The following assessment of BBB integrity was performed with IgG WB, albumin WB and AIM. A significant reduction of the BBB disruption was detected with all the aforementioned techniques. The comprehensive details can be found in the original publication [5].

To avoid the necessity to perform more than one method to quantify BBB disruption and to be able to choose the most valid approach, a correlation of the results of IgG WB, albumin WB and AIM was performed. Afterwards, the temporal and financial expenditure of the three techniques were compared to determine the most valid and efficacious way to define BBB integrity.

## 3. Materials and Methods

### 3.1. Albumin Western Blot

This method was used in the original publication out of which the results were taken for correlation [5]. Dissected cortices and basal ganglia from mouse brains were homogenized with a RIPA buffer (25 mM Tris pH 7.4, 150 mM NaCl, 1% NP-40, 0.1% SDS) containing 4% proteinase inhibitor (cOmplete protease inhibitor cocktail, Sigma Aldrich, Germany) and sonified for 10 s. After centrifugation at 15,000× *g* for 30 min at 4 °C, supernatants were used for a bicinchoninic acid (BCA) protein assay and subsequent WB analysis. The lysates were mixed with a 2 × SDS-PAGE loading buffer (final concentration: 60 mM Tris, pH 6.8, 10% beta-mercaptoethanol, 5% SDS, 10% glycerol) at 95 °C for 10 min. 20 µg of the total protein was loaded on the gel, electrophoresed and transferred to a nitrocellulose membrane. After blocking for 30 min with a blocking buffer (5% nonfat dry milk, PBS, 0.05% Tween-20), the membranes were incubated with the primary antibody at 4 °C overnight at the following dilutions: anti-albumin mAb 1:1000 (ab106582, Abcam, Cambridge, UK) and anti-actin mAb 1:1,000,000 (#A5441, Merck, Darmstadt, Germany). After washing with PBST (PBS, 0.05% Tween-20), the membranes were incubated for 1 h with HRP-conjugated donkey anti-chicken IgG (Dianova, Hamburg, Germany) or donkey anti-mouse IgG (Jackson ImmunoResearch, Cambridge, UK) at a dilution of 1:10,000 and were finally developed using Western Lightning Plus-ECL kit (PerkinElmer, Waltham, MA, USA) with the ChemiDoc Touch imaging system (Bio-Rad, Hercules, CA, USA). The albumin concentration served as comparison parameter among the single animals and groups.

### 3.2. IgG Western Blot

This method was used in the original publication out of which the results were taken for correlation [5]. The first steps until the administration of the blocking buffer were analogous to those of method 3.1. After blocking for 30 min with a blocking buffer (5% nonfat dry milk, PBS, 0.05% Tween-20), the membranes were incubated for 1 h with donkey anti-mouse IgG (Jackson ImmunoResearch, Cambridge, UK) only at a dilution of 1:10,000, and were thereafter developed using a Western Lightning Plus-ECL kit (PerkinElmer, Waltham, MA, USA) with the ChemiDoc Touch imaging system (Bio-Rad, Hercules, CA, USA). The IgG concentration served as comparison parameter among the single animals and groups.

### 3.3. Albumin Immunofluorescence Staining and Intensity Measurement

This method was used in the original publication out of which the results were taken for correlation [5]. For immunofluorescence staining against albumin, cryo-embedded coronal brain sections (2 mm) were cut into 10-µm-thick slices, fixed with aceton for 15 min and blocked for 60 min in 10% BSA in PBS containing 1% goat serum to prevent unspecific binding. A chicken polyclonal anti albumin antibody (#ab106582, Abcam, Cambridge, UK, dilution 1:200) was applied over night at 4 °C in PBS containing 1% BSA. After washing, the brain slices were further incubated with Alexa 647-labeled goat anti-chicken antibody (#ab150175, Abcam, Cambridge, UK) at a dilution of 1:100 in 1% BSA in PBS for 60 min. For the staining of DNA (cell nuclei), the sections were embedded in ProLong Gold Antifade Mountant with DAPI dye (P36931, Thermo Fisher, Waltham, MA, USA) and Aqua-Poly/Mount (#18606, Polysciences, Warrington, PA, USA). A slice of 0.5 mm anterior from the bregma was used for evaluation, and albumin detection was performed under a microscope (Leica DMi8 equipped with the DFC 3000 G camera control and LAS X software (Leica, Wetzlar, Germany)). To quantify the albumin intensity of the ipsilesional as well as the contralesional hemispheres, the immunofluorescence staining was analyzed with ImageJ (Wayne Rasband, National Institutes of Health, USA; http://imagej.nih.gov/ij; access date: 18 December 2020; Java 1.8.0_112, 64-bit). The multi-color images were split into single channels, and the albumin channel of interest converted from color images into a 16-bit grayscale image before proceeding. Afterwards, a duplication of the image was created. One of the two copies was left as it was, the other adjusted to a binary image. To create a binary image, the following instructions have to be performed: Image => Adjust => Threshold. All structures of interest can be highlighted, the background processed. Only black and white pixel intensities will remain. To start the analysis, the following settings were performed in the measurement setup: “set measurements” => “redirect to”, whereby the image that was still greyscale and not binary was chosen. The binary image was selected and the button “analyze particles” used. We chose mm as calibration unit. After pressing the “OK”-button the intensity scores are provided. The same procedure was performed independently for the ipsilateral and contralateral hemisphere of each mouse, and subsequently their ratio was calculated. The ratio served as comparison parameter among the single animals and groups.

### 3.4. Time Scheduling

To determine the average time to perform the different methods for quantifying BBB leakage, the times were taken, and, in each case, three well-trained senior researchers conducted the albumin and IgG WB as well as the AIM. The period to obtain the brain tissue itself was not measured, as it was equivalent for all three methods. The time period was taken to process the samples of eight animals, which comprises four WB samples for each animal (ipsilateral and contralateral cortical as well as ipsilateral and contralateral basal ganglial specimens) as well as two consecutive brain slices of each animal for AIM. The methods were performed as depicted above. Times were taken both for the whole processes comprising the active steps of the procedure as well as the preparation and post-processing and as exclusive hands-on-time during which the researcher is bound to the task.

### 3.5. Cost Breakdown

The purchase prices for the chemicals, antibodies and consumption material were determined for customary quantities at the beginning of the year 2021, as quoted on the homepages of the respective manufacturers (Table S1). The quantities used were normed to the procession of eight animals (2 × 4 WB samples and 2 brain slices each). Expenses for technical equipment were not included in this study.

### 3.6. Statistical Analysis

For the statistical analysis, the GraphPad Prism 8 software package was used. Data were tested for Gaussian distribution with the D’Agostino-Pearson omnibus normality test and then analyzed with the Pearson product-moment correlation coefficient as a measure of strength of linear association between two variables, denoted by r. Values for r between 0.5 and 1.0 are proposed to indicate a strong positive association. Probability values of *p* < 0.05 were considered to indicate statistically significant results.

## 4. Results

### 4.1. The Ex-Vivo Methods to Quantify BBB Leakage Correlate Significantly

**Figure 1:**
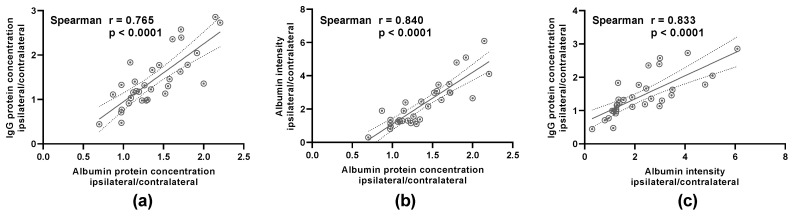
The outcomes of the three different methods to evaluate the blood brain barrier (BBB) disruption—IgG Western blot (WB), albumin WB and albumin intensity measurement (AIM)—correlate significantly. Pearson’s correlation analysis was performed to analyze the correlation between (**a**) the IgG protein concentration and the albumin protein concentration, (**b**) the immunohistochemically determined albumin intensity and the albumin protein concentration as well as (**c**) the IgG protein concentration and the immunohistochemically determined albumin intensity. The samples were sourced out of the penumbra region of the ipsilesional hemispheres as well as the corresponding contralesional brain regions. The reciprocal correlation between the three different methods was highly significant (*n* = 32).

The data used as basis for the following correlations can be found in the original publication: [5]. To quantify the BBB leakage, either (1) the ratio of ipsilateral to contralateral IgG, as determined with WB, (2) the ratio of ipsilateral to contralateral albumin, again as determined with WB, or (3) the ratio of ipsilateral to contralateral albumin intensity, as assessed with immunofluorescence staining, was analyzed. The outcomes of the respective methods were compared between the three different approaches, showing a highly significant correlation between each other (*p* < 0.0001) (Figure 1).

### 4.2. IgG Western Blot Is the Most Time- and Cost-Efficient Method to Quantify BBB Leakage

**Figure 2:**
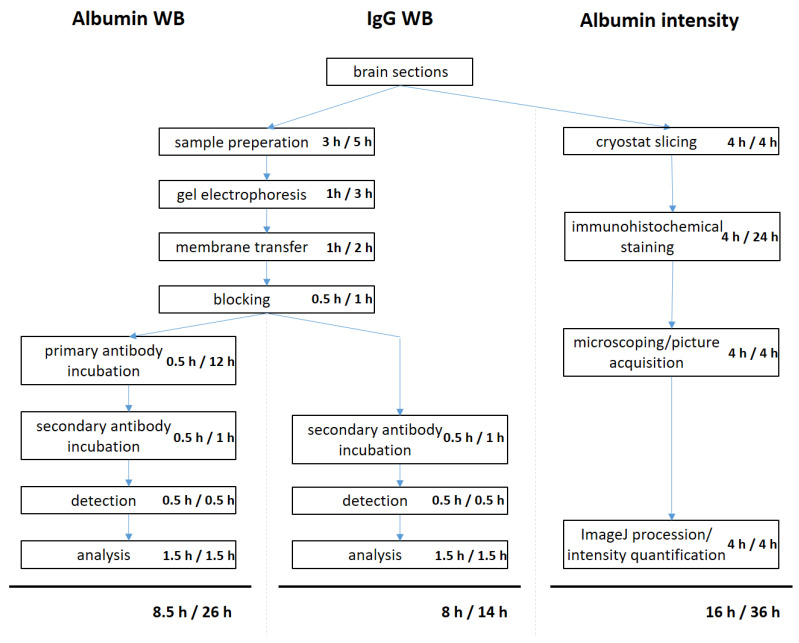
The IgG WB is the less time-consuming method in comparison to albumin Western Blot and AIM. The time investment for the single steps of the three different methods to evaluate BBB disruption was identified for a group size of eight mice. The first listed time period depicts the hands-on time, during which the scientist is tied to the respective procedure. The second time period comprises the active steps of the procedure as well as the preparation and post processing steps, if needed.

In the analysis of the times required to perform the single steps of the three different methods to define BBB integrity, a superiority of the IgG WB could be demonstrated both in the hands-on-time as well as the complete gross processing time. Valuable time was saved in comparison to the albumin WB, as the primary antibody incubation time could be skipped. AIM includes several time-consuming hands-on work steps that amounted to the longest processing times (Figure 2).

With regard to the financial aspects of the three different procedures, a similar pattern emerged. For the IgG WB, the fewest financial expenditures were incurred for chemicals, antibodies and consumption materials, while the AIM caused the highest expenses (Table 1). A detailed list of the matter of expenses can be found in Table S1.

## 5. Study Significance

Given the continuous advances in neurologic research, with—for example—the growing insights into the interactions of thrombotic and inflammatory processes at the vascular interface within IS, we need reliable animal models to base the future translational research on the present progress. To reconcile this with increasing demands of ethics and animal welfare, we need to design animal studies with as few individuals as possible. In the case of IS research no extra animals need to be sacrificed in order to quantify BBB leakage. With a single 10-µm thick coronal brain slice or small amounts of brain lysates, which would probably be processed for further protein analysis anyway, the performance of albumin WB, IgG WB and/or the measurement of albumin immunofluorescence intensity are easily achievable and reliable as long as in-vivo measurements are dispensable.

We were the first to match the outcomes of the three aforementioned techniques to measure BBB integrity and could show a highly significant correlation between their respective results. Consequently, the data quality as such is not the decisive decision-making criterion for which approach to characterize the BBB should be chosen. That is why we went one step further and compared the temporal and financial aspects of the methods, too. With regard to this we could show that the IgG WB is not only the quickest to perform but also the cheapest technique. With that, it is straightforward to economize future BBB studies within neurovascular and neuroinflammatory research with the use of the IgG WB.

## Figures and Tables

**Table 1:** The IgG WB is the cheapest method to determine BBB integrity. Prices were determined for *n* = 8 in Euro on 27 January 2021.

	Albumin WB	IgG WB	AIM
chemicals	56.33	56.33	43.33
antibodies	40.03	1.52	91.22
consumption material	16.65	15.73	3.51
**total cost**	**113.00**	**73.57**	**138.17**

## Data Availability

Any additional data will be provided upon reasonable request.

## Supplementary Materials

The following are available online at https://www.mdpi.com/2409-9279/4/1/23/s1, Table S1: Detailed cost breakdown for chemicals, antibodies and consumption material of albumin WB, IgG WB and AIM.
