# Supplementary material for: Validity and Efficacy of Methods to Define Blood Brain Barrier Integrity in Experimental Ischemic Strokes: A Comparison of Albumin Western Blot, IgG Western Blot and Albumin Immunofluorescence
Source: Methods Protoc. 2021 Mar 23;4(1):23. doi: 10.3390/mps4010023 (PMC8005953; doi:10.3390/mps4010023)
Supplement: Supplementary file 1 [file mps-04-00023-s001.pdf]

methods and protocols

MDPI

Validity and Efficacy of Methods to Define Blood Brain Barrier Integrity in Experimental Ischemic Strokes: A Comparison of Albumin Western Blot, IgG Western Blot and Albumin Immunofluorescence

Maximilian Franke, Michael Bieber, Guido Stoll and Michael Klaus Schuhmann *

Department of Neurology, University Hospital Wuerzburg, Josef-Schneider-Str. 11,
97080 Würzburg, Germany; franke_m1@ukw.de (M.F.); bieber_m@ukw.de (M.B.); stoll_g@ukw.de (G.S.);
* Correspondence: schuhmann_m@ukw.de

Supplementary Material

Table S1: Detailed cost breakdown for chemicals, antibodies and consumption material of albumin WB, IgG WB and AIM.

Name	Supplier	Order numb	Pack size	Price [€]/ package	Used quantity in IgG WB	*Price [€] for IgG WB*	quantity in albumin WB	*for albumin WB*	Used quantity in AIM	Price [€] for AIM
				**chemicals**						
Acrylamide 30 %	Carl Roth	3029.1	1 l	64,50	7,5 ml	0,48	7,5 ml	0,48	-	-
Tris(hydroxymethyl)aminomethan	Roche	1,0709E+10	1 kg	97,92	11 g	1,08	11 g	1,08	-	-
Sodium dodecyl sulfate	Merck	1137601000	1 kg	179,20	1,5 g	0,27	1,5 g	0,27	-	-
Tetramethylethylendiamine	Merck	T9281-100ML	0,1 l	33,00	0,04 ml	0,01	0,04 ml	0,01	-	-
Ammoniumperoxodisulfate	Merck	A3678-100G	0,1 kg	38,36	0,021 g	0,01	0,021 g	0,01	-	-
Glycin	Merck	G8898-1KG	1 kg	36,96	43,2 g	1,58	43,2 g	1,58	-	-
Sodiumchloride	Th.Geyer	13671000	1 kg	10,00	0,05 g	0,01	0,05 g	0,01	-	-
Tergitol-type NP-40	Merck	74385-1L	1 l	54,31	0,045 ml	0,02	0,045 ml	0,02	-	-
Protease inhibitor cOmplete®	Roche	1,1697E+10	40 ml	331,00	0,2 ml	1,65	0,2 ml	1,65	-	-
Sertile Water	Local pharma	-	1 l	3,00	1 l	3,00	1 l	3,00	1,5 l	4,50
Phophatase inhibitor Phos Stop Eas	Roche	4906837001	20 ml	256,00	0,1 ml	5,12	0,1 ml	5,12	-	-
BCA Protein Assay Kit®	Merck	71285	0,5 l	328,00	22 ml	14,40	22 ml	14,40	-	-
Glycerin	Merck	G2025-500MI	0, 5 l	41,71	0,1 ml	0,02	0,1 ml	0,02	-	-
β-mercaptoethanol	Merck	M3148-100M	0, 1 l	11,45	0,1 ml	0,01	0,1 ml	0,01	-	-
Bromphenol blue	Merck	B8026-5G	5 g	51,30	0,005 g	0,05	0,005 g	0,05	-	-
Methanol	Local pharma	-	1 l	66,00	60 ml	3,96	60 ml	3,96	250 ml	16,50
Bovine serum albumin	Merck	-	1 kg	2430,00	-	-	-	-	1 g	2,43
Ponceau S-solution	Merck	P7170	1 l	75,51	15 ml	1,13	15 ml	1,13	-	-
Milk powder	Carl Roth	T145.2	0,5 kg	21,90	1,2 g	0,05	1,2 g	0,05	-	-
H2O2-solution 30%	Carl Roth	CP26.1	1 l	18,50	15 ml	0,28	15 ml	0,28	-	-
Octylphenoxypolyethoxyethanol	Merck	9002-93-1	0,5 l	63,90	-	-	-	-	5 ml	0,64
Enhanced Chemiluminescence Subs	Perkin Elmer	NEL 105001E/	680 ml	616,00	12 ml	10,80	12 ml	10,80	-	-
Precision Plus Protein Western C Sta	Bio-Rad	161-0376	0,25 ml	208,00	0,012 ml	10,05	0,012 ml	10,05	-	-
Precision Protein StrepTactin HRP®	Bio-Rad	161-0380	0,3 ml	162,00	0,003 ml	1,62	0,003 ml	1,62	-	-
Phopshate buffered saline 1x	Selfmade	-	1 l	4,91	100 ml	0,49	100 ml	0,49	1 l	4,91
Polysorbat 20 (Tween-20)	Merck	P9416-100ML	0,1 l	33,21	0,75 ml	0,24	0,75 ml	0,24	-	-
Pap-Pen®	Merck	Z672548	1 pc	96,40	-	-	-	-	15%	14,46
**subtotal chemicals**						**56,33**		**56,33**		**43,44**
				**antibodies**						
Chicken anti albumin	Abcam	ab106582	0,1 ml	480,00	-	-	0,0075 ml	32,00	-	-
HRP goat anti chicken	Abcam	ab6877	0,5 ml	225,00	-	-	0,003 ml	1,35	-	-
Mouse anti actin	Merck	A5441-100UL	100 µl	395,00	0,00012 ml	0,48	0,00012 ml	0,48	-	-
HRP donkey anti-mouse IgG	Dianova	715-035-150	0.5 ml	172,00	0,002 ml	1,04	0,002 ml	1,04	-	-
HRP donkey anti-mouse IgG	Dianova	715-035-150	0.5 ml	172,00	-	-	0,01 ml	5,16	-	-
Rabbit anti albumin	Abcam	ab207327	0,1 ml	555,00	-	-	-	-	0,005 ml	55,50
Alexa Fluor 488 donkey anti rabbit	BioLegend	AF594	0,5 ml	258,00	-	-	-	-	0,01 ml	10,32
ProLong Gold Antifade®	Thermo Fishe	P36930	10 ml	254,00	-	-	-	-	1 ml	25,40
**subtotal antibodies**						**1,52**		**40,03**		**91,22**
				**consumption material**						
Western-Blotting-Membranes, nitro	Merck	GE10600002	1 pc	249,00	0,01 pc	2,50	0,01 pc	2,50	-	-
Whatman paper	Hartenstein	GB33	50 pcs	179,00	0,2 pc	0,33	0,2 pc	0,33	-	-
Tubes 1,5 ml	Hartenstein	RSF1	2000 pcs	37,17	100 pcs	1,59	100 pcs	1,59	4 pcs	0,06
Tubes 2,0 ml	Hartenstein	RK2G	1000 pcs	20,46	45 pcs	0,92	45 pcs	0,92	4 pcs	0,08
Cuvettes	Eppendorf	759115	100 pcs	15,42	45 pcs	1,68	45 pcs	1,68	-	-
Falcon® 15 ml	Eppendorf	11507411	1000 pcs	117,00	5 pcs	0,58	5 pcs	0,58	2 pcs	0,23
Falcon® 50 ml	Eppendorf	10788561	1000 pcs	155,00	10 pcs	1,55	15 pcs	2,33	-	-
Plastic pipette	Hartenstein	PSB	400 pcs	29,00	8 pcs	0,58	10 pcs	0,73	6 pcs	0,04
Pipette tips	Hartenstein	FB15/FB10	3000 pcs	30,00	150 pcs	1,50	150 pcs	1,50	20 pcs	0,20
Combitips advanced®	Hartenstein	EC10	100 pcs	150,00	3 pcs	4,50	3 pcs	4,50	-	-
Microscope slides	Hartenstein	OTS	72 pcs	21,90	-	-	-	-	8 pcs	2,43
Cover slip	Hartenstein	DK50	100 pcs	5,90	-	-	-	-	8 pcs	0,47
**subtotal comsumption material**						**15,73**		**16,65**		**3,51**
**total expenses**						**73,57**		**113,00**		**138,17**

The table lists the expenses for chemicals, antibodies and consumption material as well as the total expenses for n = 8 per method. (Carl Roth: Karlsruhe, BW, Germany; Roche: Basel, Switzerland; Merck: Darmstadt, HE, Germany; Perkin Elmer: Waltham, MA, United States of America; Bio-Rad Technologies: Hercules, CA, United States of America; Abcam: Cambridge, CAM, United Kingdom; Thermo Fisher: Waltham, MA, United States of America; Hartenstein: Würzburg, BY, Germany; Eppendorf: Hamburg, HH, Germany).
